# Carbon dots–cadmium sulfide quantum dots nanocomposite for ‘on–off’ fluorescence sensing of chromium(vi) ions

**DOI:** 10.1039/d4ra00436a

**Published:** 2024-04-22

**Authors:** Anisha B. Patil, Pooja L. Chaudhary, Parag V. Adhyapak

**Affiliations:** a Centre for Materials for Electronics Technology (C-MET), (Scientific Society, Ministry of Electronics & Information Technology (MeitY), Govt. of India) Panchawati, off Pashan Road Pune 411008 India adhyapakp@gmail.com adhyapak@cmet.gov.in +91-20-25898180 +91-20-25899273

## Abstract

This work involves fluorescent probe which is composed of carbon dots (CD) and cadmium sulfide quantum dots (CdS QD) for the sensitive and selective fluorescence detection of chromium(vi) ions. The blue fluorescent carbon dots were synthesized by hydrothermal method from natural precursor apricot. The carbon dots–cadmium sulfide quantum dots (CD–CdS QD) nanocomposite was synthesized and all as-synthesized products were characterized using different characterization techniques. It showed white fluorescence under UV light which was quenched selectively in the presence of chromium(vi) ions due to the inner filter effect (IFE). The linear decrease in the white fluorescence was observed in the concentration range 2–120 μM of chromium(vi) ions with the limit of detection 2.07 μM. This is novel probe for the sensitive, selective and rapid detection of chromium(vi) ions.

## Abstract 2

This work involves fluorescent probe which is composed of carbon dots (CD) and cadmium sulfide quantum dots (CdS QD) for the sensitive and selective fluorescence detection of chromium(vi) ions.

## Graphical abstract

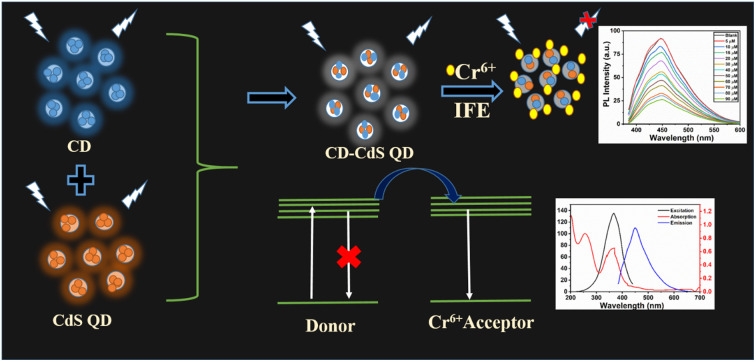

## 1. Introduction

Heavy metal ions are referred to the metal ions with higher density (>5 g cm^−3^). Heavy metal ions can be classified into essential such as Zn^2+^, Fe^2+^, Co^3+^, Cu^2+^, Mn^2+^*etc.* and non-essential such as Pb^2+^, Hg^2+^, As^3+^ and Cr^6+^. Among these, non-essential heavy metal ions are toxic, carcinogenic and hazardous to human health even at low concentrations. Therefore, there are restrictions on the usage of these heavy metal ions in different commodities led by global organizations. Moreover, the presence of these heavy metal ions in the environment, specifically in the air, soil and natural water resources are need to be tackled since these are the direct/indirect sources for human/animal intake.^1^ Therefore, it is essential to develop simple, reliable and accurate sensor to detect heavy metal ions.

Chromium is one of the widely used heavy metal ions in different industrial processes pertaining to electroplating, tanning, dying, pigmentation, coating, alloying *etc.*^2^ Out of two stable oxidation states of chromium such as trivalent (Cr(iii)) and hexavalent (Cr(vi)), the Cr(iii) is essential for human metabolism. Whereas, Cr(vi) is highly toxic, carcinogenic and can cause complicated health issues in human beings by damaging respiratory, intestinal, nervous, reproductive, immunological systems.^3,4^ The U.S. Environmental Protection Agency has recommended the concentration of Cr(vi) to be not more than 100 μg L^−1^ in drinking water.^5^ The world health organization has set the limit of Cr(vi) as 0.05 mg L^−1^ in drinking water.^6^ Therefore, the detection and tracking of Cr(vi) concentration is important. There are various methodologies already available for detection of chromium and other heavy metal ions in general such as electrochemistry,^7^ surface enhanced Raman scattering,^8^ colorimetry,^9,10^ atomic absorption spectrometry,^11^ inductively coupled plasma mass spectrometry^12^*etc.* These methodologies are cumbersome and require costly instruments and proper setup. It is desirable to have simple, rapid and sensitive technique for the detection of heavy metals. The fluorescent probe method is one of the methods which can detect heavy metal ions based on simple technique and materials *viz.* quantum dots, metallic nanocluster and organic dyes *etc.*

CdS quantum dots are well known due to its size confinement, large surface area and narrow band gap. The excellent optical and electronic properties made them to use in various applications such as fluorescent sensors, electrochemical sensors and solar cells *etc.*^13,14^ CdS QD is widely used fluorescent material for developing fluorescent probe for detection of various heavy metal ions such as Cu,^15^ Hg^16,17^ and Ag.^14^

Carbon dots have gained research interest due to their excellent luminescent properties along with low toxicity, small size, biocompatibility, chemical stability, tunable excitation emission spectra and high quantum yield.^18,19^ Owing to the excellent properties, carbon dots have emerged as an excellent material for fluorescent sensing of different heavy metal ions.^20,21^ Yang *et al.* discovered the novel test paper method for the detection of Cr(vi).^22^ Zhang *et al.* reported N-doped based carbon dots as fluorescent probe for the detection of Cr(vi) and ascorbic acid with lower limit of detection 0.30 μmol L^−1^.^23^ Omer *et al.* has reported N,P-doped carbon dots for the detection of chromium(ii).^24^

Carbon dots are good candidate for many applications due to their good optical and electrical properties and can show excitation dependent photoluminescence behaviour due to different sizes and surface defects or even sometimes the excitation independent behaviour. Similarly, CdS quantum dots also show luminescence in the visible region. The combination of any two fluorescent quantum dots is fascinating and can be used as application tool in sensing. In this report, we also got white fluorescence by combining these two compounds which is unusual. This type of observed behaviour is presumably due to the overlapping of the energy bands and the energy transfer between them when excited in UV or visible region which is discussed in detail in this manuscript.^25^ Getting white fluorescence was always been interesting due to its applications in various fields such as LEDs, tunable lasers and sensors. Generally inorganic and organic molecules can show white fluorescence due to its properties.^26^ White light can be achieved due to mixing of the two or three complementary colours by tuning the quantities.^27^ Amalgamation of these two excellent fluorescent materials may lead to material with enhanced fluorescent properties that can be used as an application tool in sensing. The composite could take advantage of optical properties of both the materials and properties like easy synthesis, low cost, fluorescence properties, stability and biocompatibility which make it useful in sensing.

Herein we report a fluorescent sensor based on carbon dots–cadmium sulfide quantum dots (CD–CdS QD) for the effective detection of Cr(vi). CD were synthesized by simple, rapid and green synthesis method. The CdS QD were synthesized by reported hydrothermal method. The nanocomposite CD–CdS QD was prepared which produces white light emission in UV light. The CD, CdS QD and CD–CdS QD nanocomposite were characterized using the different techniques like Ultraviolet visible spectroscopy (UV-Vis), Photoluminescence (PL) spectroscopy, Field emission transmission mlectron microscopy (FETEM), X-ray photoelectron spectroscopy (XPS) and Fourier transform infrared (FTIR) spectroscopy, zeta potential analysis and lifetime decay measurements. We report a turn off fluorescent sensor for selective and sensitive detection of Cr(vi) in the range 2–120 μM. The white fluorescence of nanocomposite was decreased linearly in the range 2–10 μM with the detection limit of 2.07 μM. The fluorescence quenching in the presence of Cr(vi) occurs due to the inner filter effect as UV-Vis spectra of Cr(vi) overlaps with the excitation/emission spectra of probe and there is no change in the fluorescence lifetimes of the sample before and after the addition of Cr(vi).

## 2. Experimental section

### 2.1 Materials

Apricots were purchased from local market. Cadmium(ii) chloride anhydrous, mercury(ii) chloride, potassium dichromate were purchased from spectrochem ltd. Nickel(ii) chloride hexahydrate, copper(ii) chloride dihydrate were purchased from Fischer scientific. Iron(iii) chloride, barium(ii) nitrate, lead(ii) acetate trihydrate, magnesium(ii) sulphate heptahydrate, Chromium(iii) oxide, Cobalt(iii) chloride and citric acid were purchased from Qualigens. Iron(ii) chloride tetrahydrate was purchased from Thomas baker, zinc(ii) chloride and sodium hydroxide pellets from Merck chemicals, manganese sulphate monohydrate from SD fine chemical ltd., arsenic(iii) oxide from Alfa aesar. Thioacetamide was procured from lobachem ltd. Sodium phosphate dibasic heptahydrate and sodium phosphate monobasic monohydrate were purchased from Loba chemicals. Deionized water was used throughout the experiments.

### 2.2 Synthesis of CD

CD were prepared by single step hydrothermal method using apricot as carbon source. The detailed procedure is as follows. 2.66 g of apricot was grated and dispersed in 50 mL of DI water and soaked for 2 h. This solution was then filtered through Whatman filter paper grade 1. Out of this filtrate; 25 mL was collected and mixed with 25 mL of ethanol. This solution was transferred to 100 mL Teflon lined stainless steel autoclave and heated at 150 °C for 3 h. The obtained brown colored solution of CD was naturally cooled and again filtered through Whatman filter paper grade 1 to remove any residual impurities present. The CD were collected as a filtrate and stored for further analysis and making composite. The resulting CD brown solution showed blue fluorescence under UV light.^28^

### 2.3 Synthesis of CdS QD

The CdS QD were prepared by hydrothermal method earlier reported elsewhere.^15^ Firstly, 0.25 mmol CdCl_2_and 0.5 mmol citric acid were dissolved in 50 mL DI water. After 15 min of stirring, the pH value was adjusted to 10 using 1 M NaOH and then 0.0625 mmol thioacetamide was added to the solution under continuous stirring. The reaction mixture was stirred for 30 min and then transferred to 200 mL Teflon lined stainless steel autoclave. The autoclave was heated at 120 °C for 2 h. The obtained yellow colored solution was used as it is for further analysis and making composite. The obtained solution showed orange fluorescence under UV light.

### 2.4 Preparation of CD–CdS QD nanocomposite

The CD–CdS QD nanocomposite was prepared by mixing appropriate quantities (experimentally optimized) of CD and CdS QD to get white fluorescence. For this purpose, 100 μL of CD solution was taken into the beaker and to this solution 4 mL of CdS QD solution was added. This mixture was stirred for about 10–15 min. The resultant yellowish colored composite solution exhibited white fluorescence under UV light.

### 2.5 Characterization

The as prepared CD, CdS QD and CD–CdS QD nanocomposite had been characterized using different characterization techniques. Optical absorption properties were studied using UV-visible spectrometer (Jasco V-570). Optical emission studies and sensing experiments were carried out using Fluorolog Horiba jobinyvon at different excitation wavelengths. The FTIR analysis was done using the instrument IRAffinity-1S by Shimadzu. The liquid sample was coated on the glass substrate for this analysis. The morphology was observed using field emission transmission electron microscope (FETEM) Model: JEOL 2200FS operated at 200 keV electron energy. The samples were dispersed in ethanol and later coated on copper grid for the analysis. The X-ray photoelectron spectroscopic study was carried out by using model XPS, ESCA-3000, VG Scientific Ltd at pressure1 × 10^−9^ torr. The fluorescence decay analysis was done using the Fluorescence Endinburg instrument FS5. Zeta potential measurement was carried out by using Horiba PSA DLS SZ-100-Z2.

### 2.6 Quantum yield (QY) measurement

The QY of the as-synthesized CD, CdS QD and CD–CdS QD nanocomposite were measured by diluting the sample in DI water. The UV-Vis and fluorescence spectra were recorded using 10 mm quartz cuvette. Quinine sulphate in 0.1 M [H_2_SO_4_] was used as a standard reference for CD, CD–CdS QD and 0.1 M rhodamine 6G for CdS QD. The QY of quinine sulphate is 0.54 and rhodamine 6G is 0.95. The following equation was used to evaluate the QY.
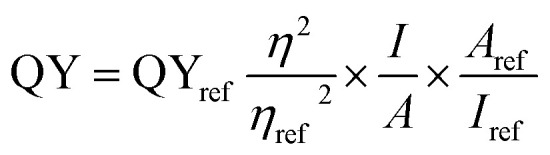
where QY_ref_ is the QY of the reference material (0.54 for quinine sulphate and 0.95 for the rhodamine 6G), *η* is the refractive index of the solvent, *η*_ref_ is the refractive index of reference, *A* is the absorption at the given wavelength, and *I* is the integrated fluorescence emission intensity. The fluorescence QY of CD at *λ*_ex_ = 375 nm and CD–CdS QD at *λ*_ex_ = 350 nm were calculated to be 3.18%, and 1.04% respectively and the integrated luminescence intensity of CD and CD–CdS QD was compared to that of standard quinine sulfate. The fluorescence QY of CdS QD at *λ*_ex_ = 375 nm was calculated to be 5.08% with rhodamine 6G as a reference.

### 2.7 Metal ion sensing

The various fluorescent based materials are explored nowadays and used for heavy metal ion sensing according to the literature survey. The selectivity of fluorescent CD–CdS QD nanocomposite was checked with 15 different metal ions like Cu^2+^, Ni^2+^, Zn^2+^, Mn^2+^, Mg^2+^, Fe^2+^, Fe^3+^, Ba^2+^, Hg^2+^, Pb^2+^, Cd^2+^, Cr^6+^, Cr^3+^, Co^3+^ and As^3+^. For this purpose, 1 mM stock solutions of all these interfering metal ions were prepared by using respective salts in DI water. To check the selectivity, 200 μL of composite sample was mixed with 2 mL of phosphate buffer (pH 5.8) and 2 mL of 50 μM solution of different metal ions solutions. The nanocomposite showed good selectivity towards the Cr^6+^ ions with fluorescence quenching. The fluorescence measurements were taken and spectra were recorded at excitation wavelength of 350 nm.

## 3. Results and discussion

### 3.1 Characterization of CD, CdS QD and CD–CdS QD

**Fig. 1:**
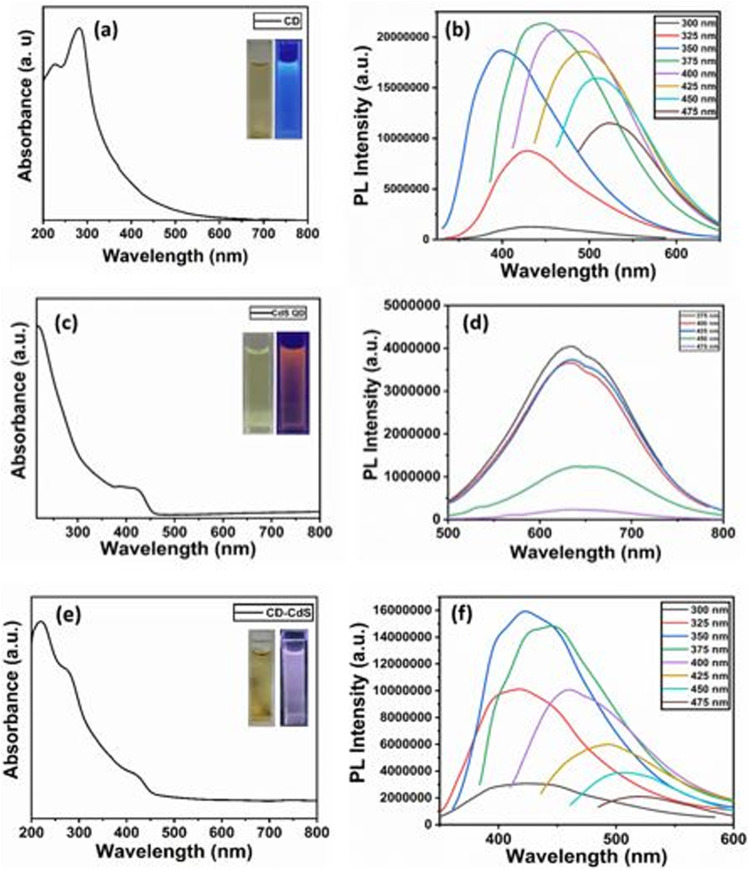
UV-visible spectra of (a) CD (c) CdS QD (e) CD–CdS QD (inset: solution under day light (left) and UV light (right)) and photoluminescence spectra of (b) CD (d) CdS QD (f) CD–CdS QD.

The optical absorption of as-synthesized products have been characterized using UV-Vis spectrometer. Fig. 1a displays optical absorption spectrum of CD. It shows absorption peaks at 227 nm and 282 nm which can be corresponded to π → π* transition of C=C and n → π* transitions of C=O respectively.^29^ The results are in accordance with the literature. The CD solution when observed under UV light show strong blue fluorescence (inset Fig. 1a).

The UV-visible spectra of CdS QD (Fig. 1c) shows peak at 424 nm. The peak at 424 nm confirms the presence of CdS quantum dots. The blue shift is observed than the CdS bulk material which shows peak at 522 nm.^30^ The CdS QD show orange fluorescence under UV light (inset Fig. 1c).

The UV-visible spectra of CD–CdS QD (Fig. 1e) nanocomposite shows peaks at 220 nm and 278 nm which confirms the presence of CD and peak at 422 nm confirms the presence of CdS QD in the nanocomposite. In case of nanocomposite, the CD absorption peaks observed at 220 nm and 278 nm are slightly blue shifted than the bare CD which confirms the formation of nanocomposite. The nanocomposite shows white fluorescence under UV light (inset Fig. 1e).

The Fig. 1b shows the photoluminescence spectra of CD which shows the excitation dependent photoluminescence behavior.^29^ The sample was excited at different excitation wavelengths from 300 nm to 475 nm with the increment of 25 nm. The red shift is observed with the increase in the excitation wavelength. The highest PL intensity is observed at 447 nm when excited at 375 nm.

The PL spectra of CdS QD (Fig. 1d) shows peak at 633 nm. The CdS QD PL spectra shows excitation independent behaviour.^15^ The sample was excited from 375 nm to 475 nm, however there is no shift. The highest intensity is recorded when excited at 375 nm.

The PL spectrum of CD–CdS QD nanocomposite (Fig. 1f) shows excitation dependent photoluminescence behaviour. The nanocomposite was excited at different wavelengths from 300 nm to 475 nm and the red shift is observed. The highest intensity is observed at 422 nm when excited at 350 nm.

**Fig. 2:**
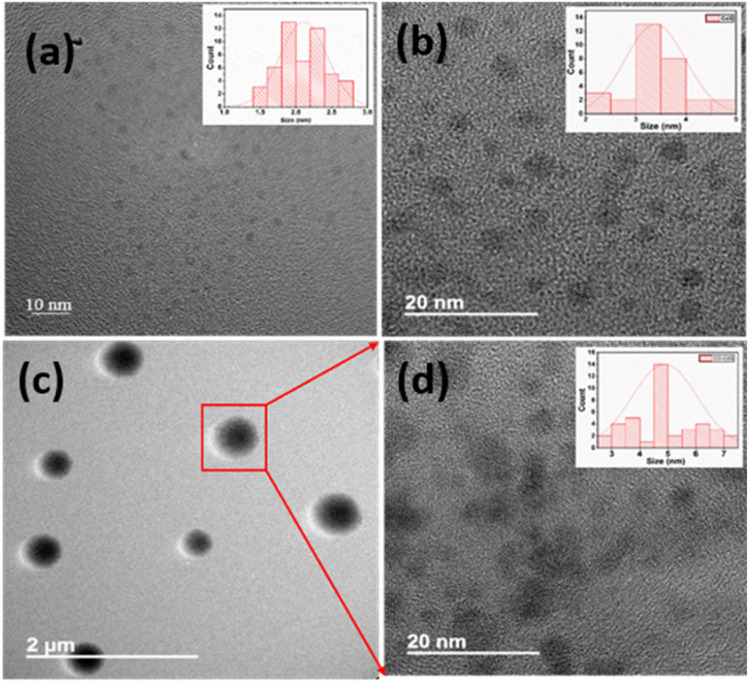
FETEM images of (a) CD, inset: particle size distribution of CD (b) CdS QD, inset: particle size distribution of CdS QD (c) CD–CdS QD nanoclusters (d) CD–CdS QD nanocomposite focused image, inset: particle size distribution of CD–CdS QD.

**Fig. 3:**
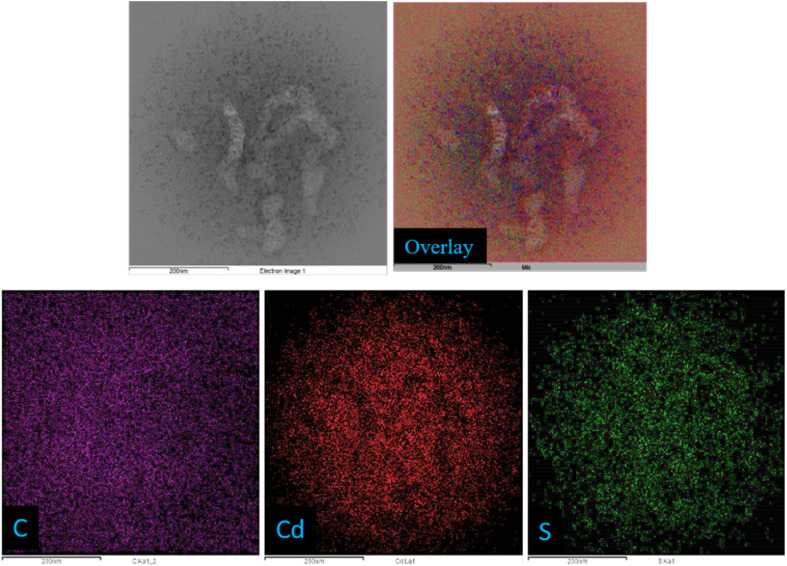
Representative HAADF-STEM and the corresponding EDX mapping of elements C (violet), Cd (red), S (green) and the overlay of CD–CdS QD nanocomposite.

The morphology and the size of as-synthesized products have been investigated using field emission transmission electron microscopy (FETEM) analysis. The FETEM photomicrographs of CD (Fig. 2a) show uniformly dispersed CD with spherical morphology. The average particle size observed is about 1–3 nm in diameter. The particle size distribution is shown as inset Fig. 2a. The FETEM image of CdS QD (Fig. 2b) also shows the uniformly distributed spherical particles of CdS with average particle size between 2–4 nm diameters (inset Fig. 2b). In case of CD–CdS QD nanocomposite (Fig. 2c), the clusters are observed which are formed due to simultaneous collective agglomeration of CD and CdS QD. The clusters are spherical in shape with average size around 200–500 nm. The focused single cluster (Fig. 2d) clearly shows the soft spherical agglomeration of CD and CdS QD with size 4–7 nm in diameter (inset Fig. 2d). The presence of CD and CdS QD in these clusters was further confirmed by HAADF-STEM and corresponding EDX elemental mapping of these nanoclusters is presented in Fig. 3. The high-angle annular dark field scanning TEM (HAADF STEM) mode of selected cluster shows the formation of spherical cluster of size around 500 nm which is covering the full image. The cluster is observed to be made up of soft agglomerated nanoparticles of CD and CdS QD with size less than 10 nm. The elemental mapping images also show presence of C, Cd and S in the selected sphere.

**Fig. 4:**
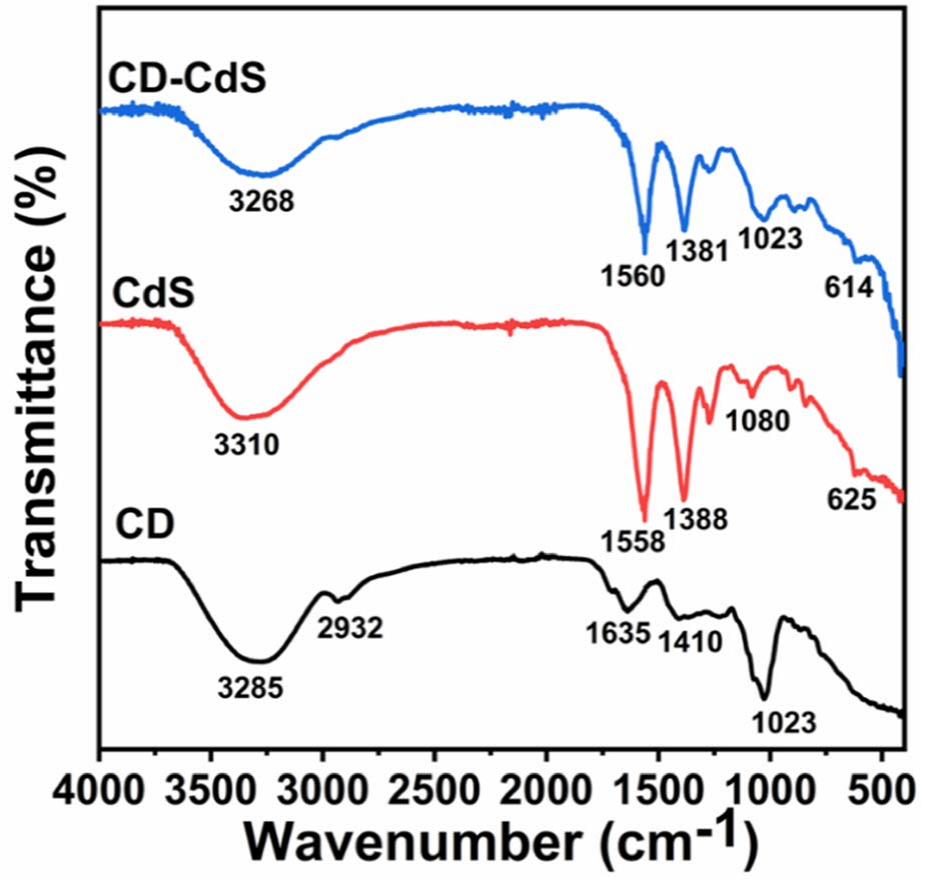
FTIR spectra of CD, CdS QD and CD–CdS QD.

The Fig. 4 depicts the FTIR spectra of CD, CdS QD and CD–CdS QD nanocomposite. The broad band observed at around 3250–3310 cm^−1^ in all the products can be assigned to O–H stretching vibrations. This O–H stretching vibration band has been observed to be shifted to lower wavenumbers in case of nanocomposite than individual CD and CdS QD. This implies that O–H might have contributed for composite formation by providing the binding site. The C–H stretching vibration is observed only in case of CD *c.a.* 2932 cm^−1^. The bands at 1635 cm^−1^ and 1410 cm^−1^ are observed in CD because of C=O and COO^−^ groups present at the surface of CD.^31^ The bands *c.a.* 1558 cm^−1^and 1388 cm^−1^are observed in CdS QD can be assigned to asymmetrical and symmetrical stretching of carboxylate functions of citrate.^32^ These bands are also present in case of nanocomposite with slightly reduced intensities. The C–O stretching band is observed in all the products at around 1050 cm^−1^. The peak at 625 cm^−1^ in pristine CdS QD shifted to 614 cm^−1^ in the nanocomposite which is the characteristics peak of CdS QD which confirms its presence in the nanocomposite. The change in the wavenumbers suggests that, there can be interaction between the functional groups of CD and CdS QD.^33^

**Fig. 5:**
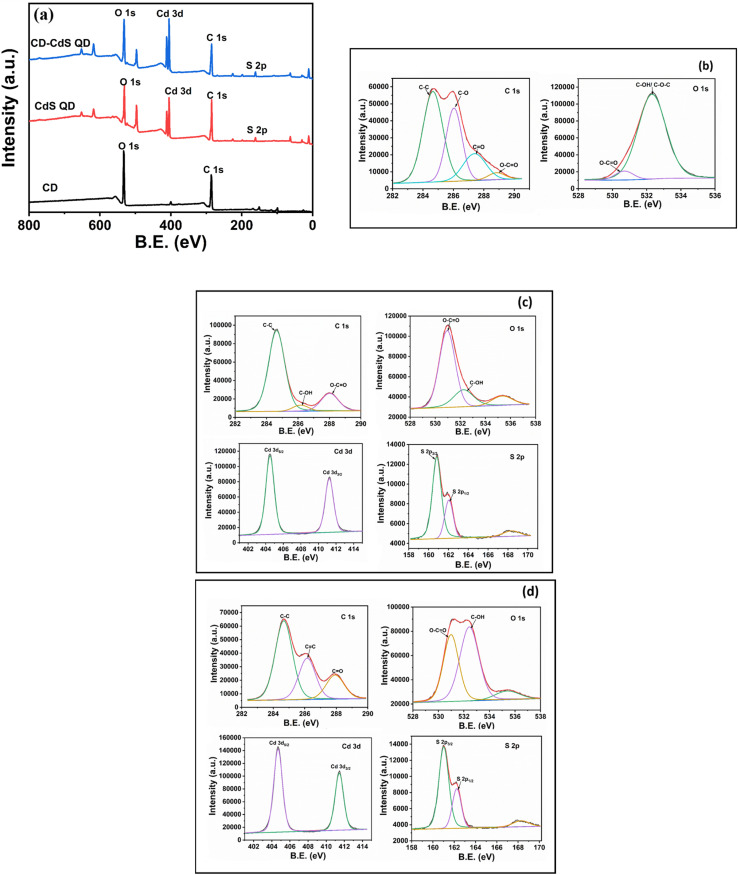
(a) XPS survey scan of CD, CdS QD and CD/CdS QD (b) XPS scans of CD (c) XPS scans of CdS QD (d) XPS scans of CD/CdS QD.

The X-ray photoelectron spectroscopy (XPS) analysis was performed to study the surface chemical composition of the as-synthesized CD, CdS and CD–CdS QD. The Fig. 5a presents the survey scan of all as-synthesized products which show peaks corresponding to pertinent present elements in respective products. The Fig. 5b presents the XPS scans for CD which exhibit C 1s and O 1s spectra. The C 1s spectra is deconvoluted into four peaks at 284.6 eV, 286 eV, 287.4 eV, and 288.6 eV which are attributed to C–C, C–O, C=O, O–C=O bands respectively.^34^ The XPS spectra of O 1s of CD is having peaks at 530.6 eV, 532.4 eV corresponding to O–C=O and C–OH/C–O–C bands respectively.^35^

The XPS scan for pristine CdS QD (Fig. 5c) indicates Cd 3d XPS spectra with peaks at 404.4 eV and 411.2 eV which are attributed to Cd 3d_5/2_ and Cd 3d_3/2_ which confirms the presence of Cd^2+^ in the pristine CdS QD. The XPS spectra of S 2p of bare CdS QD (Fig. 5c) indicates peaks at 160.8 eV, 162 eV and 168 eV due to S 2p_3/2_, S 2p_1/2_ and oxidized form of S respectively.^15^

**Fig. 6:**
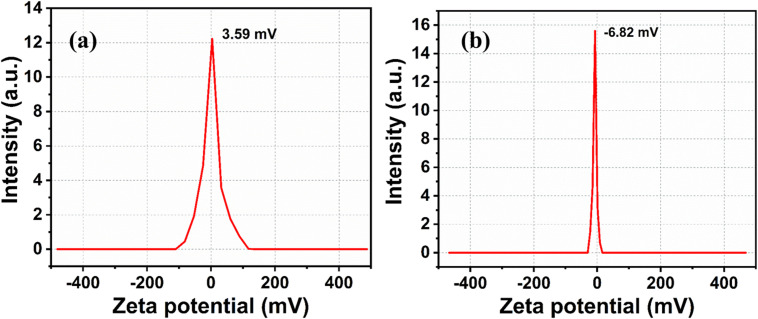
Zeta potential of (a) CD and (b) CdS QD.

In case of nanocomposite, the Cd 3d XPS spectra (Fig. 5d) have peaks at 404.7 eV and 411.4 eV which are due to Cd 3d_5/2_ and Cd 3d_3/2_ respectively and the S 2p XPS spectra of CD–CdS QD (Fig. 6d) shows peaks at 161 eV and 162.2 eV which are attributed to S 2p_3/2_ and S 2p_1/2_ respectively. The peak at 168.2 eV is assigned to oxidized form of S *i.e.* SO_3_^2−^ and SO_4_^2−^ on the external surface. The presence of Cd 3d and S 2p in the XPS of nanocomposite confirms the presence of the CdS in the nanocomposite. As the positive shift can be seen in the binding energies of Cd 3d and S 2p in the composite than the pristine CdS QD. The positive shift and change in the intensity suggest a decrease in the electron density of CdS which may be due to the interaction between CdS QD and CD. There can be electron transfer from CdS QD to CD.^36,37^ In the C 1s XPS spectra (Fig. 5d) of the nanocomposite, the peaks at 284.6 eV, 286.1 eV and 288 eV are due to the C–C, C–OH and O–C=O bands respectively which confirms the presence of CD in the nanocomposite.^38^ The O 1s spectra of CD–CdS QD (Fig. 5d) have peaks at 530.9, 532.4 and 535.3 which are attributed to C–OH, O–C=O bands and auguer peak of Na respectively.^15,39^ The XPS results are well in agreement with the FTIR data.

The Fig. 6a and b shows the zeta potential measurements of CD and CdS QD. The zeta potential values of CD and CdS QD are 3.59 mV and −6.82 mV respectively. It is concluded that CD and CdS QD can be associated due to electrostatic attraction between their surface functional groups as both are oppositely charged molecules.

The interaction between the CD and CdS QD is confirmed from the zeta potential, FTIR and TEM studies. The electrostatic interaction is confirmed between them from the zeta potential measurements. The TEM images shows the increased size of nanocomposite which is due to the soft agglomeration of both the samples which is also confirmed from the HAADF STEM and EDX mapping. The presence of all the pertinent elements is confirmed from the mapping as well as XPS spectra. The particle size distribution also shows the increased size in case of composite than the bare CD and CdS QD.

### 3.2 Fluorescence stability

**Fig. 7:**
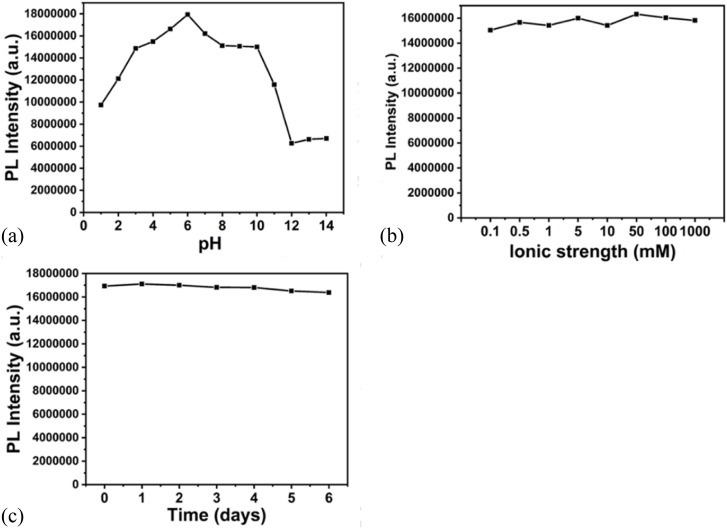
Effect of (a) pH^1–14^ (b) ionic strength (NaCl conc 0.1 mM–1 M) (c) time on the fluorescence intensity of the nanocomposite.

The fluorescence stability was checked with the effect of pH, ionic strength and the stability time. The Fig. 7a shows the effect of pH on the fluorescence of nanocomposite. It is observed that the intensity is higher in the range of pH 4–7. The fluorescence is lower in more acidic and alkaline medium. The highest fluorescence intensity is observed at pH 5.8. Fig. 7b shows the changes in the fluorescence intensity on changing the NaCl concentration in the range 0.1 mM–1 M. There is no significant change in the intensity, on changing the ionic strength, indicating the feasibility of probe in high salt concentration. Fig. 7c shows the fluorescence intensity of the composite over the period of 6 days. It is observed that the fluorescence is stable for long period.

### 3.3 Detection of Cr^6+^

**Fig. 8:**
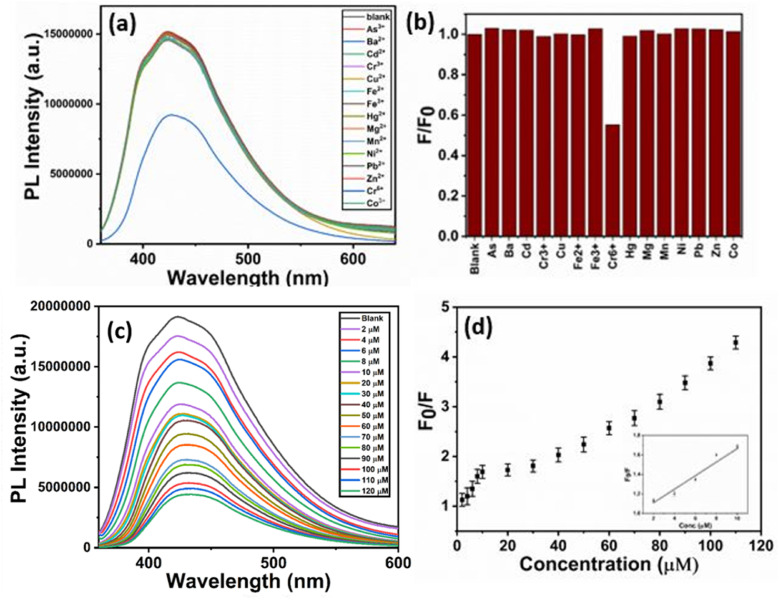
Selectivity of the probe in presence of different metal ions (a) fluorescence study (b) bar diagram of *F*/*F*_0_*vs.* metal ions (c) fluorescence study of probe in presence of different concentrations of Cr^6+^ ions (d) relative fluorescence response of CD–CdS QD (*F*_0_/*F*) *versus* concentration of Cr^6+^.

The selectivity of CD–CdS QD fluorescent probe was checked with 50 μM solutions of 15 different metal ions *viz.* Cu^2+^, Ni^2+^, Zn^2+^, Mn^2+^, Mg^2+^, Fe^2+^, Fe^3+^, Ba^2+^, Hg^2+^, Pb^2+^, Cd^2+^, Cr^6+^, Cr^3+^, Co^3+^ and As^3+^. The Fig. 8a presents the photoluminescence spectra which show fluorescence intensity of probe in the presence of different metal ions. It was observed that the white fluorescence was quenched in the presence of Cr^6+^ ions and the fluorescence change was negligible in the presence of 50 μM solutions of other metal ions which showed the strong selectivity towards Cr^6+^ ions than other metal ions. Fig. 8b shows bar diagram of fluorescence response of CD–CdS QD (*F*/*F*_0_) for different metal ions. This exhibited ‘turn off’ sensor as the fluorescence of sensing probe was quenched.

The sensitivity study of probe for Cr^6+^ ions was done with different concentrations in the range 2–120 μM and the emission spectra were recorded. The fluorescence intensity was decreased linearly with the increasing concentrations of Cr^6+^ ions in the range 2–10 μM (Fig. 8c). The relative fluorescence response of CD–CdS QD (*F*_0_/*F*) *versus* concentration of Cr^6+^ is shown in the Fig. 8d where *F*_0_ represents the fluorescence intensity in absence of Cr^6+^ and *F* represents the fluorescence intensity in presence of Cr^6+^. A steady decline in fluorescence intensity of probe with increasing concentration of Cr^6+^ was observed. The response time of probe to react with heavy metal ions was less than 1 minute which is quite low and better for detection of heavy metals. The correlation coefficient 0.965 proves the linear relationship. The limit of detection was 2.07 μM based on 3*σ*/slope method.

Table 1 shows the comparison of the CD–CdS QD probe with the already reported literature for Cr^6+^ detection using different precursors. The limit of detection is comparable with the earlier reported work and it is lower than detection limits set by WHO and U.S. Environmental Protection Agency. As compared to other reports, the sensing method used is simple, rapid and sensitive. The starting material used for CD synthesis is green. The synthesis of nanocomposite is easy, rapid and this nanocomposite material is rarely used before for this application. The fluorescence of as-synthesized products is stable for long time. The response time of the probe is less than 1 minute which is one of the advantages for heavy metal detection. The composite is showing white fluorescence which can only get by adding the complementary fluorescent colors which is quite interesting and useful in the LEDs, lasers, lamps. So, the material can be used for WLEDs in future. This suggests that the CD–CdS QD can be efficient towards Cr(vi) detection.

**Table 1:** Comparison of the present study with the previously reported reports

Sensing probe for Cr^6+^	Linear range	LOD	Ref.
N-doped CDs	5–200 μM	4.16 μM	6
CDs	2–300 μM	0.4 μM	40
CDs	0–0.1 M	10 μM	18
CDs from shrimp shells	0–150 μM	0.1 μM	41
N-doped CDs	0.08–1 mM	0.14 mM	42
CDs derived fromPoria cocos polysaccharide	1–100 μM	0.25 μM	43
Co-doped CDs	5–125 μM	1.17 μM	44
CdS QDs	0.016 to 0.260 μM	16 nM	13
CD-SiO_2_	20–500 nM	1.3 nM	45
N-CD-CTAC	0.5–100 μM	40 nM	46
CD–CdS QD	2–10 μM	2.07 μM	Present work

### 3.4 Quenching mechanism

**Fig. 9:**
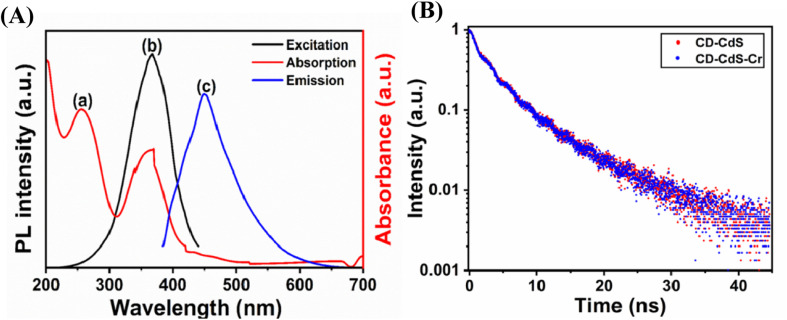
(A) (a) Absorbance of chromium (red line) (b) excitation of nanocomposite (black line) (c) emission of the nanocomposite (blue line) (B) lifetime decay curve of composite in absence of chromium(vi) ions (red) and presence of chromium(vi) ions (blue).

The fluorescence quenching by the heavy metal ion involves many possible mechanisms like inner filter effect (IFE), static quenching, dynamic quenching and forster resonance energy transfer (FRET) *etc.*^1^ Here, the fluorescence intensity of the nanocomposite was quenched in the presence of Cr(vi) ions. The possible mechanism was checked. The IFE required the good spectral overlap between the absorption band of the absorber and the excitation and/or emission band of the fluorophore.^47,48^ As shown in the Fig. 8a, the Cr(vi) ions exhibit three absorption bands at 256 nm, 361 nm and 440 nm. The CD–CdS QD nanocomposite shows excitation band at 366 nm and emission band at 449 nm. As observed from the Fig. 9A, there is good spectral overlap between the absorption of absorber (red line) and the excitation (black line) and emission band (blue line) of the fluorophore. Cr(vi) ions can absorb the excitation as well as the emission light of the CD–CdS nanocomposite. It was believed that, IFE is responsible for the fluorescence quenching.^2,6,49^ The lifetime of the composite was measured in absence (black) and presence (blue) of Cr(vi) ions as shown in Fig. 9B. There was no significant change in the lifetime which can confirm the quenching was due to the IFE.

The possibility of FRET and dynamic quenching mechanism was ruled out as there was no change in the lifetime. There was no change in the absorption of the composite in the presence and absence of the Cr(vi) ion which excluded the possibility of the static quenching mode which indicates that there is no complex formation in the composite with the heavy metal.^1^ Therefore, the possible mechanism was IFE for the quenching.

## 4. Conclusions

The carbon dots (CD) and cadmium sulfide quantum dots (CdS QD) were successfully synthesized *via* hydrothermal method. The white fluorescent CD–CdS QD nanocomposite was prepared by simple mixing of the individual entities in appropriate quantities. The CD and CdS QD are associated due to the electrostatic attraction. The sensing response of the nanocomposite was checked with different heavy metal ions solution which showed good selectivity towards the Cr^6+^ ions. The white fluorescence was decreased linearly with increasing concentration of Cr^6+^ from 2–10 μM in the range of 2–120 μM. The limit of detection and the correlation coefficient was 2.07 μM and 0.965 respectively. The quenching of the fluorescence was probably due to the inner filter effect (IFE) which was confirmed from the overlap of absorbance of fluorophore and excitation/emission of the probe. The developed sensing method is simple, sensitive, selective and rapid for the detection of Cr^6+^ions.

## Author contributions

Anisha B. Patil – material synthesis, characterization, sensing measurement, formal analysis and writing draft. Pooja L. Chaudhary – Material synthesis, characterization and sensing measurement. Parag V. Adhyapak – supervision, review and editing draft, project administration.

## Conflicts of interest

There are no conflicts of interest to declare.

## Supplementary Material
